# Presenting Visual Acuities in a Referral Eye Center in an Oil-Producing Area of Nigeria

**DOI:** 10.4103/0974-9233.53866

**Published:** 2009

**Authors:** Joseph M. Waziri-Erameh, Afekhide E. Omoti

**Affiliations:** From the Department of Ophthalmology, University of Benin Teaching Hospital, PMB 1111, Benin City, Nigeria

**Keywords:** Blindness, Niger delta, Nigeria, Visual acuity

## Abstract

**Objectives:**

To determine the pattern of presenting visual acuities at an eye center in the Niger Delta region of Nigeria.

**Study Design:**

Retrospective chart review

**Methods:**

A retrospective review of patient records attending a private referral eye center providing services for company patients and the general public in the region. Information was obtained from computerized medical records of 6533 patients who attended the center for various eye concerns in a 5-year period (January 1998 to December 2002).

**Results:**

A total of 6533 patients were seen in this 5-year period of which 2472 (37.8%) were company patients and 4061 (62.2%). were private patients. There were 3879 males (59.4%) and 2654 females (40.6%). A visual acuity of 6/6 or better was seen in 50.8% of the patients. In 76.6% of patients, a visual acuity of 6/18 or better was recorded. There were 21.4% of patients in the low vision group. Bilateral blindness occurred in 2.1% of patients. Monocular blindness occurred in 3.7% of patients. Low vision occurred in 16.9% of company patients and 24.1% of private patients. Bilateral blindness occurred in 0.9% of company patients and 2.7% of private patients, while monocular blindness occurred in 1.2% of company patients and 5.2% of private patients. The main ocular problems were refractive error, glaucoma, conjunctivitis, headaches, ocular trauma, retina and related pathologies, cataract, uveitis, pterygium and corneal problems.

**Conclusion:**

The incidence of low vision and blindness is high in the oil-producing area of the Niger Delta region of Nigeria. Low vision and blindness were more common in private patients than in company patients.

## INTRODUCTION

There are about 180 million people worldwide with visual disability, out of which between 40 and 45 million are blind^1^ and over 110 million have low vision.^2^ About 90% of the world's blind live in the developing world^3^ including Nigeria. Nigeria has a blindness rate of 1%. This 1% varies greatly from about 1.5% in the far North of the country and about 0.5% in the South.^4^

Warri is the main town of south Midwest region of Nigeria. South Midwest region of Nigeria has a population of about 2.5 million people and about half of the people are in the riverine area. It is a major oil-producing area of Nigeria but the story is that of neglect and lack of amenities. There is no tertiary health institution in the region and there is very little documentation of the disease pattern in the region. Thus there are a very few published studies on ocular health. This clinical-based retrospective inspection of presenting visual acuity of eye patients at DDS Eye Center, Warri, south Midwest region of Nigeria is perhaps the first in the region on visual acuity.

## MATERIALS AND METHODS

This study was done at the Dr Daljit Singh Eye Center, a private referral eye center providing services for referred company patients and the general public in the region. Information was obtained from the computerized medical records of 6533 patients who attended the center for various eye concerns in a 5-year period (January 1998 to December 2002). During this period, 2472 patients were company-referred patients from private medical practices and medical clinics of oil companies/multinationals in the region, while 4061 patients were from the general public (private) all over the region. We noted the visual acuity for each patient at presentation in both groups (company and private) from 6/6 and better to no light perception (NLP). We looked at the age and sex distribution and also a panoramic overview of the ocular problems of the patients. We also grouped the visual acuity findings into normal vision, low vision and bilateral and monocularly blind.

## RESULTS

The total number of patients in this 5-year study was 6533. Company patients were 2472 (37.8%), while private patients were 4061 (62.2%). Total number of males was 3879 (59.4%) while total number of females was 2654 (40.6%). Majority of the patients were in the age range 20–50 years accounting for 67% of all the patients seen. The ages were not recorded in 247 patients (3.8%). Company patients were 1563 males (63.2) and 909 females (36.8); while private patients were 2226 males (54.8%) and 1835 females (45.2%) (Table 1).

**Table 1:** Age, sex and group distribution of the 6533 patients

Age (Years)	Sex		Total	Total Percent	Patient Group	
				
	Males	Females			Company	Private
0–10	255	245	500	7.7	141	359
11–20	371	527	898	13.8	300	598
21–30	667	550	1217	18.6	381	836
31–40	814	465	1288	19.7	639	649
41–50	923	325	1248	19.1	691	557
51–60	376	185	561	8.6	199	362
61–70	164	169	333	5.1	31	302
Above 70	140	101	241	3.7	18	223
Unknown age	169	87	247	3.8	72	175
Total	3879	2654	6533	100	2472	4061

Visual acuity of 6/6 or better was seen in 50.8% of the total number of patients seen. The company patients had 54.7% VA 6/6 or better, while 48.3% of private patients had VA 6/6 or better. In functional vision groupings, 76.6% of the patients had VA 6/18 or better (normal); the company patients had 82.2%, while the private patients had 73.1%. In the low vision group, 6/24 to count fingers (CF) 21.4% of the total patients were in this category, 16.9% in the company patients and 24.1% in the private patients. Bilateral blindness was seen in 2.1% of the total number of patients, 0.9% of company patients and 2.7% of private patients. Monocular blindness occurred in 3.7% of total patients, 1.2% of company patients and 5.2% of private patients (Tables 2–4). The spectrum of the top ten ocular problems were refractive error, glaucoma, conjunctivitis, headaches, ocular trauma, retina and related pathologies, cataract, uveitis, pterygium and corneal problems (Table 5).

**Table 2:** Summary of presenting best visual acuity of the 6533 patients

VA	Company patients	Private patients	Total	Percent
6/6 and better	1351	1965	3316	50.8
6/9	308	449	757	11.6
6/12	216	296	512	7.8
6/18	157	260	417	6.4
6/24	188	405	593	9.1
6/36	121	254	375	5.7
6/60	80	200	280	4.3
3/60 (CF)	29	119	148	2.3
HM	14	66	80	1.2
LP	6	31	37	0.6
NLP	2	16	18	0.3
Total	2472	4061	6533	100

CF = Count Fingers; HM = Hand Movement; LP = Light Perception; NLP = No Light Perception

**Table 3:** Functional visual acuity groupings (a, b, c)

**Table 3a:** Age and group distribution of the 5002 patients with Normal/Satisfactory Vision (6/18 and better)

Age (Years)	Company patients	Private patients	Total	Percent	

				(*n* = 5002)	(*n* = 6533)
0-10	82	132	214	4.3	3.3
11-20	263	548	811	16.2	12.4
21-30	311	777	1088	21.8	16.7
31-40	551	606	1157	23.1	17.7
41-50	602	480	1082	21.6	16.6
51-60	157	200	357	7.1	5.5
61-70	22	102	124	2.5	1.9
Above 70	8	44	52	1.0	0.8
Unknown	36	81	117	2.3	1.8
Total	2032	2970	5002	100	73.1

**Table 3b:** Age and group distribution of the 1396 patients with Low vision-(6/24,6/36,6/60,3/60)

Age (Years)	Company patients	Private patients	Total	Percent	

				(*n* = 6533)	(*n* = 6533)
0–10	15	32	47	3.4	0.7
11–20	42	114	156	11.2	2.4
21–30	72	182	254	18.2	3.9
31–40	85	122	207	14.2	3.2
41–50	92	137	229	16.4	3.5
51–60	68	141	209	15.0	3.2
61–70	13	125	138	9.9	2.1
Above70	27	108	135	9.7	2.1
Unknown	4	17	21	1.5	0.3
Total	418	978	1396	100	21.4

**Table 3c:** Age and group distribution of the 135 patients with Bilateral Blindness (HM, LP, NLP)

Age (Years)	Company patients	Private patients	Total	Percent	

				(*n* = 135)	(*n* = 6533)
0–10	3	6	9	6.7	0.1
11–20	2	8	10	7.4	0.2
21–30	5	6	21	15.6	0.3
31–40	-	11	11	8.1	0.2
41–50	7	7	14	10.4	0.2
51–60	3	19	22	16.3	0.3
61–70	-	21	21	15.6	0.3
Above70	1	22	23	17.0	0.4
Unknown	1	3	4	3.0	0.1
Total	22	113	135	100	2.1

**Table 4:** Age and group distribution of the 242 patients with uniocular blindness

Age (Years)	Company patients	Private patients	Total	Percent	

				(*n* = 135)	(*n* = 6533)
0–10	2	9	11	4.5	0.2
11–20	2	11	13	5.4	0.2
21–30	2	27	29	12.0	0.4
31–40	6	27	33	13.6	0.5
41–50	7	18	25	10.3	0.4
51–60	2	35	37	15.3	0.6
61–70	3	43	46	19.0	0.7
Above70	4	40	44	18.2	0.7
Unknown	2	2	4	1.7	0.1
Total	30	212	242	100	3.7

**Table 5:** Classification of diagnosis of the 6533 patients

Diagnosis	Frequency	Percent
Refractive error/presbyopia	4318	66.1
Open angle glaucoma/ocular hypertension	997	15.3
Conjunctivitis	906	13.9
Headaches	828	12.7
Trauma	825	12.6
Retinal (313)/macular (312)/optic nerve problem (69)	694	10.6
Cataract	578	8.9
Uveitis	482	7.4
Pterygium	422	6.5
Corneal opacity (18)/ulceration (172)	280	4.3
Lid Problems	162	2.5
Squint	83	1.3
Hyphema	69	1.1
Couching	54	0.8
Routine eye examination	52	0.8
Miscellaneous	72	1.1

## DISCUSSION

The oil-producing region of Midwest Niger Delta region of Nigeria is under-served in amenities including health services and these contribute to the restiveness associated with the region. There is no tertiary health institution in the region and published studies on ocular health are very few, if any. This clinical-based retrospective study on presenting visual acuities in DDS Eye Center Warri (the major city in the region) is perhaps the first in the region. Many studies related to visual acuity are in the areas of blindness, low vision/visual impairment or in the eye problems being studied and perhaps none on presenting visual acuities.

The condition of visual disability is more common in developing countries.^1^ Nigeria, Africa's most populous nation shares in this burden. It is estimated that about 1% of Nigerians are blind.^4^ Cataract, glaucoma, onchocerciasis and trachoma are important causes of blindness in the North; while trachoma and onchocerciasis are replaced in the South by ocular trauma and complications of ocular trauma. In our panoramic view of ocular problems in this study (Southern Nigeria), glaucoma, cataract, ocular trauma/complications were among the top ten causes of visual impairment.

Benin City is the capital city of the northern part of Midwest region of Nigeria (Edo state). It has a well-established Federal University and a University Teaching Hospital. There are numerous publications on ocular health in Benin City but the same is not true of Warri, 100 km to the south of Benin City where this study took place. Review of ocular literature about Benin City and other centers in Nigeria show few articles directly related to presenting visual acuity at eye clinics and hospitals and the community. In addition, few studies showed breakdown or analysis of visual acuity. The study comparable to the current report is that published by Akpalaba^5^ who studied visual acuity and low vision in 1305 patients of an eye camp in Benin City. He found 92% had satisfactory vision, 5.7% low vision and 2.3% blind. This was in an eye camp population. In this clinical-based study, satisfactory vision was 76.6% in the total patient population, 82.2% in Company-referred patients and 73.1% in private patients. Low vision was seen in 21.4% of the total number of patients, 16.9% in company patients and 24.1% in private patients. We also found bilateral blindness of 2.1% in total patients, 0.9% company patients and 2.7% in private patients. Omoti^6^ in a study of causes of blindness in Benin City (eye clinic review) reported bilateral blindness in 7.5%, while 22.1% had mono-ocular blindness. Nwosu^7^ in a study of ocular problems of young adults in rural Nigeria found bilateral blindness in 0.7% and mono-ocular blindness in 1.7%. Ajaiyeoba and Fasina^8^ found bilateral blindness in 1.22% and low vision in 2.08% of those screened. While the studies by Nwosu and Ajaiyeoba can be labeled as community-based, those of Omoti, Akpalaba and the current study are clinical-based studies. Akpalaba obtained his records from an eye camp setting. The results of blindness and low vision in clinical-based studies already have selection bias and will not represent true community-based prevalence of blindness, low vision and normal/satisfactory vision because only cases with eye problems are found mainly in the eye clinics. This is also true of eye camps patronized mainly by poor people with eye problems that are unable to afford clinic fees. It is therefore not surprising that Akpalaba in an eye camp study had more prevalence of blindness of 2.3% compared to 2.1% in this study. Omoti^6^ reported the prevalence of blindness of up to 7.5% at the eye clinic department of University of Benin Teaching Hospital. The reasons for this very high prevalence may be explained by the aggregation of poor patients from many nearby states to a Federal eye center with much lower fees than a comparatively more expensive eye center as DDS Eye Center. The smaller sample size may also be contributory; 1698 patients compared to 6533 from DDS Eye Center. Ajaiyeoba and Fasina^8^ found bilateral blindness is high from age 60 years and above. In this study, majority of the bilateral blind are above 50 years in the general patient population and especially in the private patient category (more reflective of the community) than the much more affluent company patient category. This age factor may be due to the blinding conditions being age related like senile cataract, glaucoma and macular degenerations and agrees with the findings reported by others.^9^,^10^ Alakija^11^ found that taxi drivers aged between 20 and 40 years constituted about 31.7% of taxi drivers with unsatisfactory vision. This is also similar to our finding. The age group 20-40 years accounted for 33% of those with low vision. About 4% of the patients had no recorded ages. The reasons for this include illiteracy, difficulty with communication and perhaps poor diligence of the record clerk. We found a monocular blindness of 3.7% in this study group. This is higher than the 1.4% found by Ajaiyeoba in a population-based survey.^8^ The much higher prevalence in this study may be due to the high incidence of ocular trauma seen in this restive region. There was inter-ethnic war for three years in the 5 years of this study amidst military occupation and also frequent clashes between the youths and contractors/government forces.

There were a slightly greater numbers of men than women 59.4 to 40.6% (ratio 1.5:1). This may be due largely to the men being more economically empowered than the women. The ratio is even higher 1.7:1 in the company than in private patients since majority of those who have lucrative oil company employments are men. More men attending eye clinics than woman is similar to the findings by other workers in Nigeria.^6^,^10^ The men are usually the breadwinners and poor vision in breadwinners is treated with greater urgency and funds are thus more urgently budgeted for males getting solution to eye problems.

Blindness (bilateral and monocular) in the company group was much lower than in the private patients group, 0.9 to 2.7%, a ratio of 1:3. This may be explained by the higher socio-economic status of the company patients, free medical care provided for them by their companies and the unlikeliness for them/dependants to instill harmful cocktail of herbal extracts and other toxic substances unto the eyes when they have minor ocular conditions such as conjunctivitis. These harmful brews (mostly alkaline) easily melt off the cornea leading to corneal ulcers, opacities and endophthalmitis.^12^ Analysis of the diagnosis of these patients showed the ten most common ocular problems in the region (Table 5). This is similar to what is prevalent in other regions in southern Nigeria. Particularly high are refractive errors and presbyopia, which was a problem in 66.1% of the patients. This finding is similar to that of Nwosu^7^ who found refractive errors/presbyopia to have a prevalence of 74%. The role refractive errors play in poor vision has been rightly recognized by its inclusion in vision 2020 global initiative. The other top ten eye conditions are common in developing countries. We however note that retinal and related problems; often not given much priority as in developed world, is becoming important. Ocular trauma is very common in the region because of the frequent unrests and clashes between government forces and the people or inter-ethnic wars. Though not making the top ten, couching (0.8%) is an important cause of low vision and blindness in the region. The lack of eye care centers especially in the semi-inaccessible creeks in the riverine areas makes it easy for the native/traditional cataract couchers to take advantage of these poor and helpless cataract patients. It takes as long as 5 h boat journey for some of the patients to be able to travel from their creeks to an eye clinic. In many cases they embark on the journey when the pain from complication of the couching operation becomes unbearable. Though there are attempts to take eye care to some remote communities through eye camp initiatives, it is a tip of the iceberg because only a helicopter team can reach the far recesses. Many health personnel are even afraid to cross rivers with unsafe boats and perhaps also due to fear of local pirates and waterway robbers.

In conclusion, an inspection of the presenting visual acuity of the south Midwest region of southern Nigeria has shown the clinical-based prevalence of satisfactory vision, low vision, monocular and bilateral blindness. Although, clinical-based prevalence do not reflect the true prevalence of the community because of patient selection bias, it serves as a starting point and future reference base to judge efforts at blindness eradication in the region in particular. It is our belief that the blindness prevalence is much higher than the 2.1% reported in this study. We believe it may be much higher if all the patients with ocular problems in the creeks and far recesses of the region are able to afford the cost and means to attend an eye center.

## References

[1] Marioti SP (2000). Global Initiative for the elimination of Avoidable Blindness. WHO Fact Sheet.

[2] Rosovi I (1997). Blindness and Visual Disability Part I of VII: General information. WHO Fact Sheet.

[3] Thylefors B (1998). A global initiative for elimination of avoidable blindness. Indian J Ophthalmol.

[4] World Health Organisation (1987). Data on Avoidable Blindness (update 1987).

[5] Akpalaba RU Low vision in Benin City Aetiology and age characteristics.

[6] Omoti AE (2004). Aetiology of Blindness in Benin city, Nigeria. Ann Afr Med.

[7] Nwosu SN (1998). Ocular problems of young adults in rural Nigeria. Int Ophthalmol.

[8] Fasina FO, Ajaiyeoba AI (2003). Prevalence and causes of blindness and low vision in Ogun state, Nigeria. Afr J Biomed Res.

[9] Ayanru JO (1974). Blindness in Midwestern states of Nigeria. Trop Geo Med.

[10] Olurin O (1973). Causes of Blindness in Nigeria: A study of 1000 hospital patients. West Afr Med J.

[11] Alakija W (1981). Poor visual acuity of Taxi drivers as possible cause of motor traffic accidents in Bendel state, Nigeria. Occup Med.

